# Zebrafish *ambra1b* knockout reveals a novel role for Ambra1 in primordial germ cells survival, sex differentiation and reproduction

**DOI:** 10.1186/s40659-023-00430-9

**Published:** 2023-04-28

**Authors:** Camilla Maria Fontana, Francesca Terrin, Nicola Facchinello, Giacomo Meneghetti, Alberto Dinarello, Lisa Gambarotto, Annalisa Zuccarotto, Micol Caichiolo, Ginevra Brocca, Ranieri Verin, Francesca Nazio, Oliana Carnevali, Francesco Cecconi, Paolo Bonaldo, Luisa Dalla Valle

**Affiliations:** 1 Department of Biology, University of Padua, Padua, Italy; 2 Department of Animal and Aquatic Sciences, Faculty of Agriculture, Chiang Mai University, Chiang Mai, Thailand; 3 Department of Molecular Medicine, University of Padua, Padua, Italy; 4 Department of Medicine, Anschutz Medical Campus, University of Colorado, Denver, USA; 5 Department of Biology and Evolution of Marine Organisms, Zoological Station Anton Dohrn, Naples, Italy; 6 Department of Comparative Biomedicine and Food Science (BCA), University of Padova, Legnaro, PD Italy; 7 Aquatic Diagnostic Services, Atlantic Veterinary College, University of Prince Edward Island, Charlottetown, Prince Edward Island Canada; 8 Department of Pediatric Hemato-Oncology and Cell and Gene Therapy, IRCCS Bambino Gesù Children’s Hospital, Rome, Italy; 9 Department of Biology, University of Rome Tor Vergata, Rome, Italy; 10 Department of Life and Environmental Sciences, Polytechnic University of Marche, Ancona, Italy; 11 Cell Stress and Survival Unit, Danish Cancer Society Research Center, Copenhagen, Denmark; 12 Fondazione Policlinico Universitario Agostino Gemelli IRCCS, Università Cattolica del Sacro Cuore, Rome, Italy

**Keywords:** Ambra1, PGCs, Sex differentiation, Reproduction, Zebrafish, Mouse

## Abstract

**Background:**

AMBRA1 is an intrinsically disordered protein, working as a scaffold molecule to coordinate, by protein-protein interaction, many cellular processes, including autophagy, mitophagy, apoptosis and cell cycle progression. The zebrafish genome contains two *ambra1* paralogous genes (*a* and *b*), both involved in development and expressed at high levels in the gonads. Characterization of the zebrafish paralogous genes mutant lines generated by CRISPR/Cas9 approach showed that *ambra1b* knockout leads to an all-male population.

**Results:**

We demonstrated that the silencing of the *ambra1b* gene determines a reduction of primordial germ cells (PGCs), a condition that, in the zebrafish, leads to the development of all-male progeny. PGC reduction was confirmed by knockdown experiments and rescued by injection of *ambra1b* and human *AMBRA1* mRNAs, but not *ambra1a* mRNA. Moreover, PGC loss was not rescued by injection with human *AMBRA1* mRNA mutated in the CUL4-DDB1 binding region, thus suggesting that interaction with this complex is involved in PGC protection from loss. Results from zebrafish embryos injected with murine *Stat3* mRNA and *stat3* morpholino suggest that Ambra1b could indirectly regulate this protein through CUL4-DDB1 interaction. According to this, *Ambra1*^+/−^ mice showed a reduced *Stat3* expression in the ovary together with a low number of antral follicles and an increase of atretic follicles, indicating a function of Ambra1 in the ovary of mammals as well. Moreover, in agreement with the high expression of these genes in the testis and ovary, we found significant impairment of the reproductive process and pathological alterations, including tumors, mainly limited to the gonads.

**Conclusions:**

By exploiting *ambra1a* and *ambra1b* knockout zebrafish lines, we prove the sub-functionalization between the two paralogous zebrafish genes and uncover a novel function of Ambra1 in the protection from excessive PGC loss, which seems to require binding with the CUL4-DDB1 complex. Both genes seem to play a role in the regulation of reproductive physiology.

**Supplementary Information:**

The online version contains supplementary material available at 10.1186/s40659-023-00430-9.

## Background

AMBRA1 (*Activating Molecule in Beclin-1-Regulated Autophagy Protein 1*) is a multifunctional scaffold protein whose intrinsically disordered structure allows high protein-protein interaction plasticity, resulting in its involvement in a plethora of different and critical cellular pathways including autophagy, mitophagy, apoptosis and cell cycle progression [1–10]. The protein was initially identified by its crucial role in regulating neurogenesis and neural tube closure [3, 11, 12]. While its knockout is embryonically lethal in mice [10, 11], heterozygous *Ambra1*^*+/−*^ mice are characterized by pre-diabetic conditions [13], autism-like phenotype limited to female sex [14] and higher cancer susceptibility [5].

The zebrafish genome contains two *ambra1* paralogous genes, both involved in the autophagic process as well as in neural, muscular, and cardiac development, as demonstrated by morpholino (MO) knockdown [15–17]. Since data that can be obtained by knockdown approaches are limited to the first developmental stages, we recently generated a mutant zebrafish line for each paralogous gene. *ambra1a*^*ia35*^ (called *ambra1a*^*−/−*^ hereafter) and *ambra1b*^*ia36*^ (called *ambra1b*^*−/−*^ hereafter) mutants do not display overt developmental defects, due to the activation of genetic compensation mechanisms with up-regulation of the paralogous gene remaining active [17]. Conversely, the generation of an *ambra1a*^*−/−*^ */ambra1b*^*−/−*^ mutant line was not possible as double mutants cannot survive after larval stages [17]. In agreement with the embryonic lethality of Ambra1 inactivation in the mouse models [10, 11], this result suggests that silencing of both paralogous *ambra1* genes is incompatible with life. Despite the lack of morphological differences between *ambra1a*^*−/−*^ and *ambra1b*^*−/−*^ fish lines, *ambra1b* homozygous mutants develop exclusively as males following the achievement of sexual maturity, whereas heterozygous adult *ambra1b*^+/−^ and *ambra1a* mutants do not display sex ratio alterations [17].

Considering the different effects of *ambra1a* and *ambra1b* on sexual differentiation, we decided to investigate the role of Ambra1 in this process. Sex determination in *Danio rerio* domesticated lines is controlled by a polygenic system and environmental factors, such as temperature or social cues [18]. Zebrafish gonads initially develop as a bipotential organ containing germ cells and immature oocytes. At 20–25 dpf (days post fertilization), during sex-specific differentiation, the immature oocytes of presumptive females progress through oogenesis, giving rise to adult females. Conversely, in the presumptive males, the immature oocytes undertake apoptosis, and the gonads develop as testis. Therefore, the number of oocytes and their molecular signalling are considered to play critical roles in sexual development and represent a prerequisite for ovary formation [18].

In addition, experimental reduction of primordial germ cell (PGC) number during the first day of development produces all-male progeny with normal testis, while the total ablation of PGCs generates males with somatic gonad cells organized as testis but devoid of sperm [19]. Hence, as previously demonstrated [20], ovarian development is promoted by abundance of PGCs also at early developmental stages. Moreover, some proteins, such as Dead end and Nanos3, are involved in the control of PGC survival and migration, and their silencing results in all-male development [21].

In this study, we focused our attention on the possible role of Ambra1b on zebrafish sex determination and found that the all-male phenotype of *ambra1b* mutants relies on PGC reduction during early developmental stages. Remarkably, the PGC loss could be rescued with human *AMBRA1* (*hAMBRA1*) mRNA, but not with the *hAMBRA1* mRNA mutated in the CUL4-DDB1 complex binding domain, suggesting a possible interaction with this complex in regulating PGC development. Altogether, the high expression of Ambra1 proteins in zebrafish and mouse ovaries and the impairment of zebrafish reproductive capabilities unveil a new role of Ambra1 in reproductive physiology.

## Results

### High expression of *ambra1a* and *ambra1b* in zebrafish gonads points to a role in reproduction

**Fig. 1:**
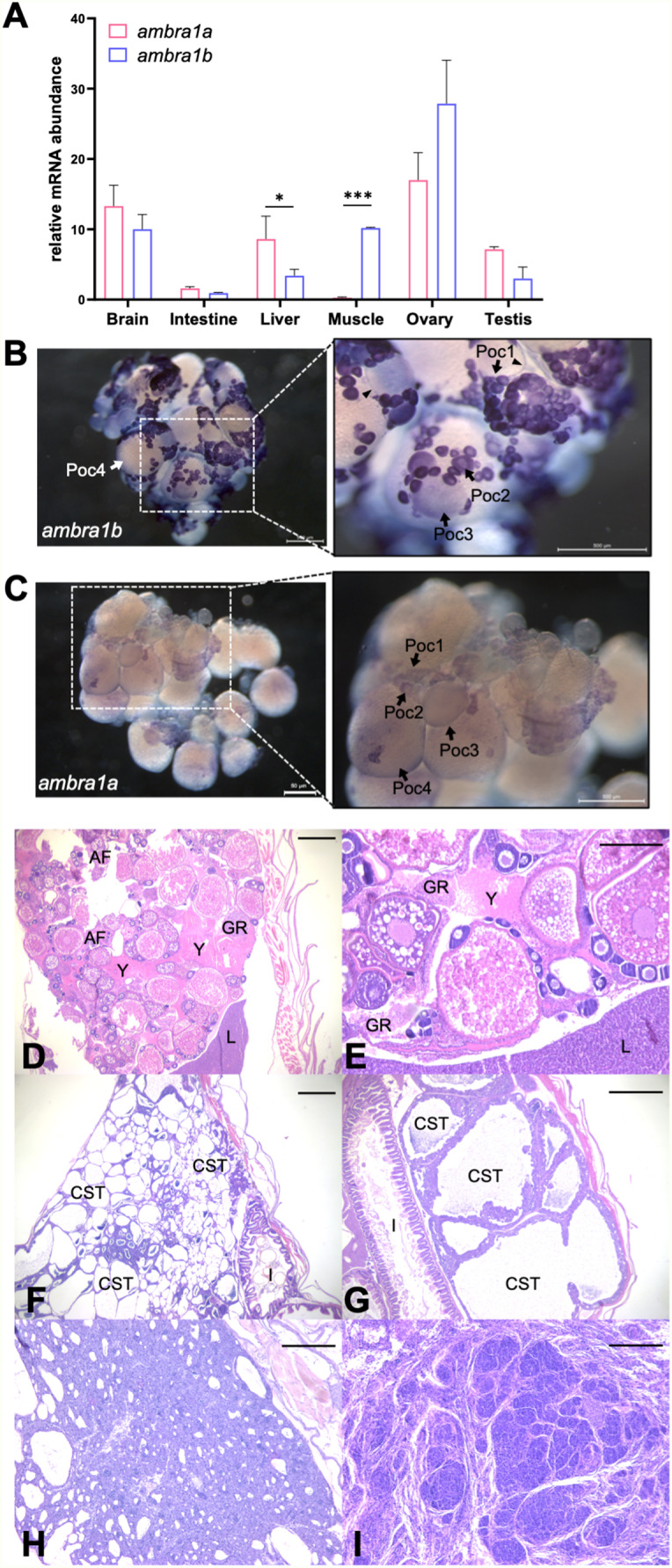
**Expression patterns of *ambra1 * mRNAs and pathological findings in the zebrafish *ambra1a*** **and* ambra1b* KO lines.** (**A**) RT-qPCR analysis of *ambra1a* **and** *ambra1b* mRNA expression in different adult zebrafish tissues. Data were generated from different biological replicates, each consisting of tissue samples from single individual (intestine, liver, ovary n = 4; brain, muscle n = 3; testis n = 2; intestine, liver, brain and muscle were a mix of female and male tissues). qPCR data were analyzed by 2^−∆CT^ method and *actb2* was selected as reference gene for normalization. Values represent the mean ± SEM. Statistical analysis was performed using Student’s t-test. * *P* < 0.05; *** *P* < 0.001. (**B**, **C**) Whole mount *in situ* hybridization with *ambra1b* (**B**) and *ambra1a* probes (**C**) in ovaries of 6-month-old WT zebrafish. Arrowheads point at somatic cells surrounding oocytes. Poc1, primary oocytes stage 1; Poc2, primary oocytes stage 2; Poc3, previtellogenic oocytes; Poc4, vitellogenic oocytes. (**D**) Representative ovary of a 18-mpf *ambra1a*^−/−^ female showing follicular degeneration and presence of yolk free in the coelomic cavity as a result of degenerated follicles (H&E; scale bar, 200 μm). (**E**) Higher magnification of panel D, showing a moderate granulomatous reaction towards proteinaceous material (H&E; scale bar, 400 μm). (**F**) Testis of a 20-mpf *ambra1b*^−/−^ male, showing diffuse cystic degeneration of the coelomic cavity (H&E; scale bar, 400 μm). (**G**) Testis of a 15-mpf *ambra1b*^−/−^ male, showing diffuse ectasia of seminiferous tubules and hyperplasia of spermatogonia lining the tubules (H&E; scale bar, 200 μm). (**H**) Testis of 18-mpf *ambra1a*^−/−^ male, displaying seminoma (H&E; scale bar, 200 μm). (**I**) Testis of a 17-mpf *ambra1b*^−/−^ male, containing an undifferentiated germ cell tumor (H&E; scale bar, 200 μm). AF, atretic follicle; CST, cystic seminiferous tubule; GR, granulomatous reaction; I, intestine; L, liver; Y, yolk.

The maintenance of both *ambra1a* and *ambra1b* paralogous genes in zebrafish and the absence of female individuals in the *ambra1b* knockout (KO) line suggested a sub-functionalization of these paralogs in zebrafish after the ancestral genome duplication [17], and possibly a different tissue-specific expression of the two paralogs. To verify this, we performed RT-qPCR on adult wild-type (WT) fish to assess the expression levels in different tissues (brain, intestine, liver, muscle, ovary, and testis). As reported in Fig. 1A, this analysis confirmed the high and comparable level of expression of both genes in the brain, in agreement with the expression and function of *Ambra1* in adult mouse brain [22, 23], whereas expression was very low in intestine and different between the two paralogs in liver and muscle, with a higher level of *ambra1a* in liver and of *ambra1b* in muscle. Furthermore, both paralogs displayed high expression levels in ovary and, although in smaller amounts, in testis, suggesting that both genes may play a role in zebrafish reproductive processes (Fig. 1A).

As both paralogous genes were highly expressed in the ovary, a whole mount *in situ* hybridization on adult WT ovaries was also performed to assess whether the two transcripts were differently localized in this organ. The analysis confirmed the RT-qPCR results, showing a higher expression of *ambra1b* transcripts (Fig. 1B,C). However, while *ambra1b* mRNA was strongly expressed in stage 1 and stage 2 primary oocytes (Fig. 1B), the *ambra1a* signal was found at lower levels in these cells but was maintained at more advanced stages of oocyte maturation such as previtellogenic and vitellogenic oocytes (Fig. 1C). *ambra1b* expression was also displayed by somatic cells surrounding previtellogenic oocytes (Fig. 1B). These data suggested a possible role for Ambra1b at the early stages of ovarian follicle development with respect to Ambra1a, whose expression seems to be maintained, although at low levels, in the maturing oocytes. This latter result is also in agreement with the higher expression of *ambra1a* mRNA in ovulated oocytes compared to *ambra1b*, as previously demonstrated [15].

We then assessed the occurrence of morphological alteration due to the genetic ablation of the two *ambra1* paralogs and carried out histological analyses on *ambra1a*^−/−^ (*n* = 14; 9 males and 5 females) and *ambra1b*^−/−^ (*n* = 30, 26 males and 4 females) individuals sacrificed at different ages, from 3 to 20 mpf (months post fertilization). WT (*n* = 19, 10 males and 9 females) were also analysed for comparative purposes.

The main pathological signs displayed by the different groups of mutants were found in individuals older than 12 mpf and, in agreement with expression data, morphological changes were mainly located in the gonads in both males and females. Pathological findings include a plethora of different changes, ranging from testicular and ovarian degeneration to inflammation and testicular neoplasia. Other phenotypic features, not related to gonads, included mild hepatic degeneration with intracytoplasmic hepatocellular vacuolar changes and skeletal muscle defects, which were constantly present, albeit at different extents, in all the examined groups except for WT subjects. The main phenotypic features of the ovarian tissue were represented by degeneration of follicles, often associated with mineralization and with an occasional inflammatory chronic granulomatous reaction towards follicular components (mainly yolk) released in the coelomic cavity (Fig. 1D,E).

Degenerative cystic changes of the seminiferous tubules were observed in two subjects, in absence of atypia (20-mpf *ambra1b*^−/−^, Fig. 1F), or only associated to a moderate dysplasia and hyperplasia of spermatogonia (15-mpf *ambra1b*^*−/−*^, Fig. 1G). Tumors of the germ cells lineage were found, ranging from well-differentiated seminomas (one 18-mpf *ambra1b*^−/−^ and one *ambra1a*^−/−^ at the same age, Fig. 1H) to undifferentiated germ cell tumor with infiltration of adjacent coelomic organs (17-mpf *ambra1b*^−/−^, Fig. 1I). Altogether, we identified one seminoma in a total of three *ambra1a*^−/−^ males and four pathological changes of the testis, two of which were cancerous, in 12 *ambra1b*^−/−^ males over one year of age.

### Both *ambra1* mutant lines display reduced reproductive capability

**Fig. 2:**
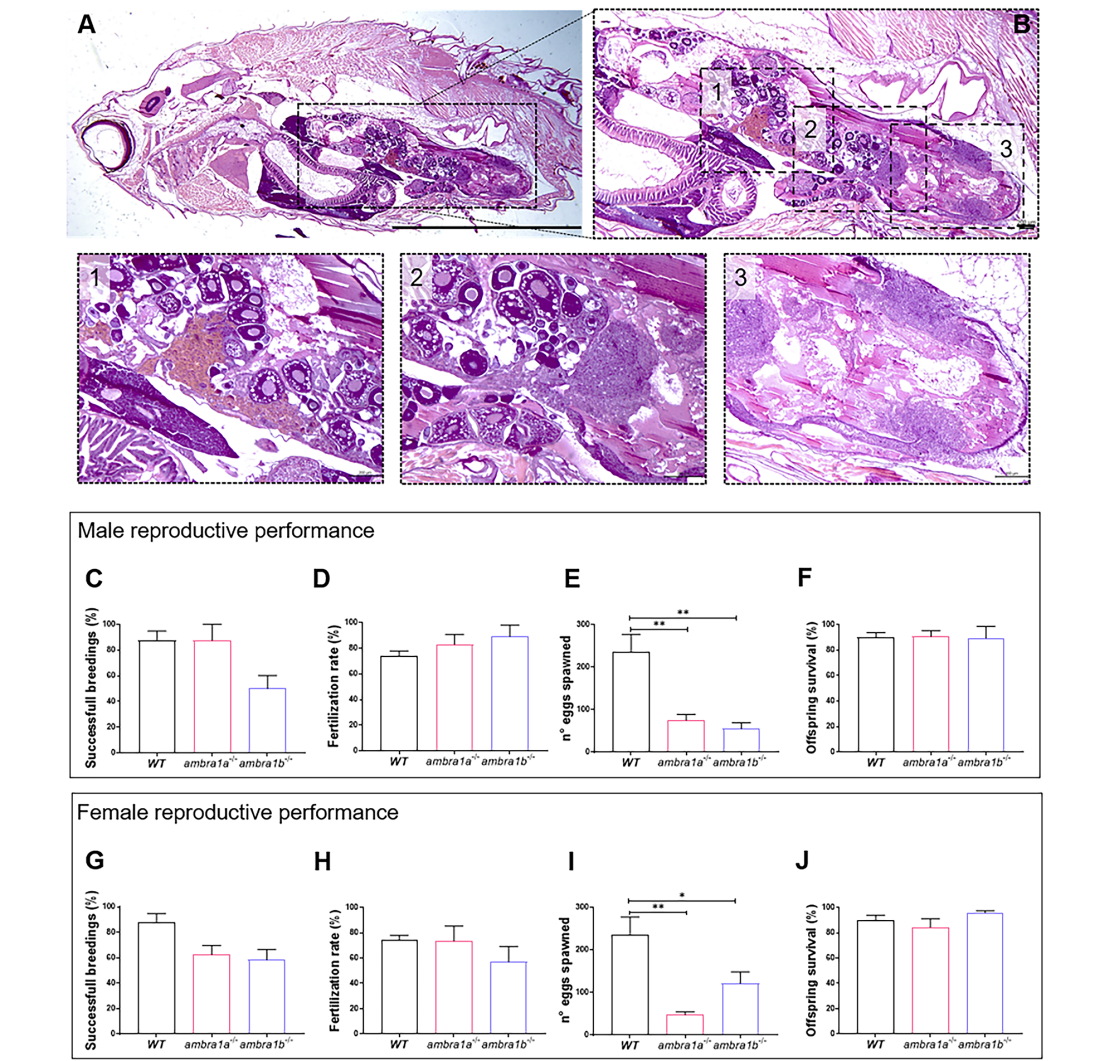
**KO of *ambra1a*** **and** ***ambra1b* reduces the reproductive capabilities of zebrafish in both sexes**. (**A**) Representative histological analysis of 16-mpf *ambra1b*^*−*/−^ female, showing H&E staining of total body sagittal section. Scale bar, 5 mm. (**B**) Magnification of the dotted area of panel A, showing the ovary region. Scale bar, 400 μm. The lower panels show higher magnifications of the respective dotted areas (1–3), corresponding to different regions of the ovary. Scale bar, 200 μm. (**C-J**) Quantification of the reproductive performance of WT, *ambra1a*^−/−^ and *ambra1b*^−/−^ males (**C-F**) and females (**G-J**). Panels C and G show the percentage of spawning success in mutant male and female tests, respectively. Panels **D** and **H** show the percentage of fertilized eggs on the total number of eggs. Panels **E** and **I** show the mean number of eggs spawned in successful breeding events. Panels **F** and **J** show the percentage of survival of the offspring at 5 dpf. Males: *ambra1a*^−/−^ n = 4; *ambra1b*^−/−^ n = 4; WT n = 4. Females: *ambra1a*^*−*/−^ n = 4; *ambra1b*^*−*/−^ n = 3; WT n = 4. Error bars indicate SEM. Statistical analysis was performed using One way ANOVA. * *P* < 0.05; ** *P* < 0.01

Although the main feature of the *ambra1b* mutant line is the lack of females, one single fish out of more than four hundred *ambra1b*^−/−^ obtained with the line propagation developed as female (less than 0,25%) and can thus be considered a “mutant escaper”. This individual (generation 1, dead at 20 mpf) displayed the classic female phenotypic features and was able to reproduce with *ambra1b*^−/−^ males in 2 out of 7 reproductive trials. The two reproductive events resulted in 53 adults, of which only 10 (19%) developed as females (generation 2). One individual of generation 2 was sacrificed at 16 mpf to perform histological analysis, which revealed the presence of all oocytes stages (Fig. 2A,B). Moreover, together with classical asynchronous development, the ovary displayed large areas of tissue degeneration, celomatic egg retention and granulomatous inflammation (Fig. 2A,B). One female from generation 2 was used to perform reproductive trials with other *ambra1b*^−/−^ males, generating generation 3, in which we found three females up to a total of 14 adult individuals. While we still do not know which compensative process allowed the development of the first escaper, it is clear that *ambra1b*^−/−^ females produce a sex-imbalanced offspring. Moreover, the ovaries of *ambra1b*^−/−^ showed clear and consistent morphological alterations, as demonstrated by histological analysis of three more female individuals (data not shown).

Considering the high expression level of both *ambra1a* and *ambra1b* in ovaries and testes, the morphological alteration in gonads and the availability of *ambra1b*^−/−^ females (from the female escaper described above), we assessed the reproductive capabilities of *ambra1a*^−/−^ and *ambra1b*^−/−^ males and females. Towards this aim, we used four 10-mpf zebrafish from both mutant lines (except for *ambra1b*^−/−^ females in which only three 10-mpf individuals were available) and compared them to WT zebrafish of the same age. Every animal was bred with WT individuals. The analysis of male reproductive capabilities showed a reduction, although not significant, of *ambra1b*^−/−^ male reproductive success compared to WT and *ambra1a*^−/−^ males (Fig. 2C). This could not be ascribed to problems with egg fertilization (Fig. 2D) or offspring survival, since these parameters were not different from WT crosses (Fig. 2F). Moreover, in both mutant lines we observed a significant reduction in the number of eggs released by their WT female partners (Fig. 2E, *ambra1a*^−/−^, p = 0,0032, *ambra1b*^−/−^, p = 0,0015).

The three *ambra1b*^−/−^ females of generation 3 obtained as described above were used to assess the reproductive abilities of *ambra1b*^−/−^ females in parallel with four *ambra1a*^−/−^ females. Reproductive capabilities were reduced in both mutant lines, although not significantly (Fig. 2G). WT males could fertilize the eggs released by mutant females and the embryos reached 5-dpf stage (Fig. 2H,J). Again, both lines showed a clear reduction in the number of eggs released per reproductive event, compared to WT (Fig. 2I, *ambra1a*^−/−^, p = 0,0024, *ambra1b*^−/−^, p = 0,0470).

### The lack of *ambra1b*^**−/−**^ females is not due to female-specific lethality and is associated with delayed gonadal development

As previously shown, *ambra1b*^−/−^ individuals, obtained from heterozygous *ambra1b* crosses, appear to be all males [17]. To exclude the possibility of female-specific lethality, we analyzed the Mendelian proportion of a breeding between female *ambra1b*^+/−^ and male *ambra1b*^−/−^. The progeny was raised to adulthood and their sex determined according to zebrafish sexually dimorphic phenotypic traits: body shape, dimorphic colour of the anal fin and abdomen, and appearance of the genital pore with the prominent genital papilla in females [18]. Among the 65 adult zebrafish that resulted from this breeding, 48% were heterozygotes with an equal proportion of male and female (ratio 1:1, female to male), and 52% were homozygotes and all males (ratio 0:2, female to male). This result had strong statistical support (χ^2^ test with a *p*-value < 0.001) and excluded the possibility of female lethality, since the ratio predicted in this case should be 1 (*ambra1b*^+/−^ females) ∶ 1 (*ambra1b*^+/−^ males) ∶ 0 (*ambra1b*^−/−^ females) ∶ 1 (*ambra1b*^−/−^ males), leading to 66% heterozygotes and 33% homozygous mutants.

Knowing that zebrafish has a window of sex determination in which the sex is decided by a system that integrates the expression of different specific genes with environmental factors [24], we performed histological analysis of gonads on 35-dpf juveniles obtained from the crossing of heterozygous female with homozygous male of both *ambra1* lines. In zebrafish, juveniles develop a “juvenile or presumptive ovary”, a bipotential gonad that histologically resembles an immature ovary. Depending on multiple signals, this structure becomes a functional ovary in about 50% of individuals. In the remaining half of individuals, the juvenile ovary degenerates, and immature oocytes undergo apoptosis while gonads start developing as testis at around 30 dpf. Then, at the 35-dpf stage, the fate decision toward the male or female phenotype has normally already been taken [25].

We analysed 12 individuals of each of the following genotypes, *ambra1a*^−/−^, *ambra1b*^+/−^ and *ambra1b*^−/−^, as well as 6 WT at the same age and classified the gonads of these juveniles as undifferentiated (without any sign of differentiation towards one or the other sex), juvenile ovary, juvenile ovary to testis transition, and ovary (Additional file 1, Fig. A1, A-D). As shown in Table 1, we could find ovaries and juvenile ovaries to testis transition in WT, *ambra1b*^+/−^ and *ambra1a*^−/−^. On the other hand, all the *ambra1b*^−/−^ individuals presented undifferentiated gonads or juvenile ovaries, but not gonads developing into female structures. These results confirmed the lack of females only in *ambra1b*^−/−^ and also pointed at a general delay in gonadal differentiation, as demonstrated by the high number of undifferentiated gonads found in heterozygous *ambra1b*^+/−^ and in homozygous *ambra1a*^−/−^ larvae. The time window of sex differentiation depends on rearing condition and diet, but since no undifferentiated gonads were found in WT larvae with this analysis, the delay appeared to be correlated with *ambra1* KO.

To confirm the all-male ambra*1b*^−/−^ phenotype, histological analysis was performed on 6 *ambra1b*^−/−^ individuals at 60 dpf. No ovaries were found, and all individuals presented a well-organized testis (Additional file 1, Fig. A1, E).

**Table 1:** Histological analysis of 35-dpf zebrafish gonads

	Undifferentiated gonad	Juvenile gonad	Juvenile ovary to testis transition	Ovary
*ambra1a* ^*−*/−^	3/12	5/12	2/12	2/12
*ambra1b* ^+/−^	5/12	3/12	2/12	2/12
*ambra1b* ^*−*/−^	6/12	6/12	-	-
WT	-	1/6	2/6	3/6

#### Table 1

Summary of the data obtained with the histological analysis of 35-dpf zebrafish sibling of the two ambra1 lines and WT samples. The numbers reported refer to the number of individuals presenting a specific type of gonad out of the total number of fish analysed.

### Ambra1b loss causes a reduction of PGCs resulting in the all-males phenotype

**Fig. 3:**
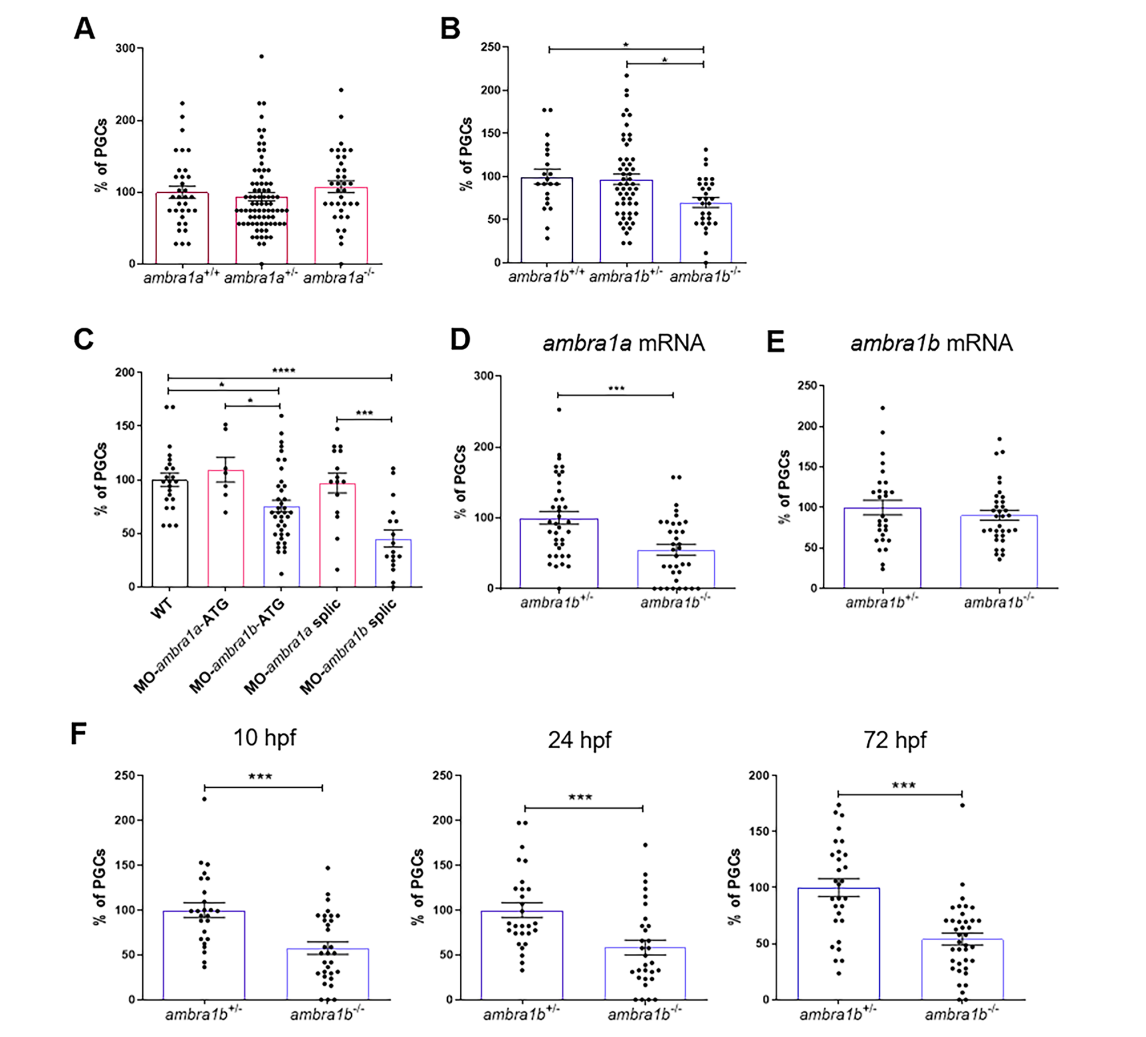
**KO of *ambra1b* leads to decreased PGC number.** (**A**, **B**) Quantification of PGCs in 10-hpf embryos of *ambra1a* (**A**), *ambra1b* (**B**) genotypes, expressed as percentage of PGCs in heterozygous and homozygous siblings compared to their WT controls. Histograms show data pooled together from three independent experiments. Only *ambra1b*^−/−^ embryos show significant reduction of PGC number (*ambra1a*^+/+^ n = 36, *ambra1a*^+/−^ n = 78, *ambra1a*^−/−^ n = 35; *ambra1b*^+/+^ n = 21, *ambra1b*^+/−^ n = 54, *ambra1b*^−/−^ n = 29). Statistical analysis was performed using One way ANOVA. *, *P* < 0.05. (**C**) Percentage of PGCs in WT embryos injected with *ambra1a* or *ambra1b* ATG MOs or splicing MOs. Analysis was performed at 10 hpf by immunohistochemistry for Vasa protein. *ambra1b* knockdown performed with both types of MO results in a significant reduction of PGCs. Histograms show data pooled together from three independent experiments (WT n = 23, MO-*ambra1a*-ATG n = 14, MO-*ambra1b*-ATG n = 41, MO-*ambra1a*-splic n = 20, MO-*ambra1b*-splic n = 20). Statistical analysis was performed using One way ANOVA. *, *P* < 0.05; ** *P* < 0.01; *** *P* < 0.001; **** *P* < 0.0001. (**D**, **E**) Results of rescue experiments by co-injection of GFP-*nos1*-3’UTR mRNA with *ambra1a* or *ambra1b* mRNA, respectively. PGC number is expressed as percentage with respect to *ambra1b*^+/−^ animals, in which the PGC number was settled as 100%. Histograms show data pooled together from three independent experiments (D: *ambra1b*^+/−^ n = 38, *ambra1b*^−/−^ n = 35; E: *ambra1b*^+/−^ n = 28, *ambra1b*^−/−^ n = 34). Statistical analysis was performed using Student’s t-test. *** *P* < 0.001. (**F**) Quantification of PGC number in *ambra1b*^+/−^ and *ambra1b*^−/−^ embryos at 10, 24,72 hpf after injection with GFP-*nos1*-3’UTR mRNA. Number of PGCs in controls (*ambra1b*^+/−^) was settled as 100%. Histograms show data pooled together from three independent experiments (10 hpf: *ambra1b*^+/−^ n = 25, *ambra1b*^−/−^ n = 31; 24 hpf: *ambra1b*^+/−^ n = 28, *ambra1b*^−/−^ n = 30; 72 hpf: *ambra1b*^+/−^ n = 29, *ambra1b*^−/−^ n = 38). Statistical analysis was performed using Student’s t-test. *** *P* < 0.001. Error bars indicate SEM.

In zebrafish, experimental reduction of PGC number at early developmental stages or their complete ablation has been shown to produce adult males with normal or sperm-free testis, respectively [19]. The loss of germ cells in the form of primary oocytes, on the other hand, occurs in juvenile ovaries during sex determination and differentiation. At this stage, the balance between low or high apoptosis levels is supposed to control oocytes survival and the maturation of a female or a male gonad [26]. While the loss of oocytes in juvenile gonads is part of the normal process of sex differentiation to testis, an early decrease of PGCs during the first developmental stages can be due to the loss of fundamental maternal or zygotic instructions, resulting in the formation of all-male adults that can suffer from fertility problems [27] Starting from these data, we sought to assess whether the absence of females in *ambra1b*^−/−^ was due to a reduction of PGCs by visualizing and counting the PGCs using whole-mount immunohistochemistry against the Vasa protein, a specific marker of germ cells [27]. This analysis was performed with 10-hpf (hours post fertilization) embryos obtained by the mating of *ambra1a* and *ambra1b* heterozygous parents. Interestingly, while we found a statistically significant reduction of the number of PGCs in *ambra1b*^−/−^ embryos compared to *ambra1b*^+/−^ (27% reduction, p = 0,011) and *ambra1b*^+/+^ (30% reduction, p = 0,0237) siblings (Fig. 3B), no reduction in PGCs was detected in the *ambra1a*^−/−^ embryos (Fig. 3A).

These results were confirmed by performing the same immunohistochemical analysis in WT embryos after knockdown of the two paralogous genes with MO-*ambra1b*-ATG and MO-*ambra1a*-ATG, as well as with the corresponding splicing MOs. The specificity of these MOs has been previously verified [15–17]. As shown in Fig. 3C, silencing of both maternal and zygotic *ambra1a* transcripts did not affect PGC number. Conversely, we obtained a statistically significant reduction of PGCs with both *ambra1b* MOs, thus confirming the data obtained with the PGC analysis of *ambra1b*^−/−^ embryos of Fig. 3B. Precisely, the injection with *ambra1b* ATG MO determined the loss of the 25% of the PGCs (Fig. 3C, p = 0,0221). Moreover, injection of *ambra1b* splicing MO determined an even more consistent PGC reduction (55% reduction, p < 0,0001), indicating that the control of PGC number cannot be only ascribed to the contribution of maternal *ambra1b* transcripts (present in the eggs of the heterozygous female used in the experiment of Fig. 3B). These results suggested that Ambra1b-dependent PGC regulation occurs later, after mid-blastula transition, and requires zygotic transcripts.

Since we did not detect any statistically significant difference in PGC number between *ambra1b*^+/+^ and *ambra1b*^+/−^, the subsequent studies were carried out, unless otherwise specified, with larvae obtained from breedings between *ambra1b* heterozygous females and homozygous males.

To better visualize PGCs in living embryos and at later stages of development, we injected GFP-*nos1*-3’UTR mRNA in the one-cell stage embryos used for the experiments. As previously demonstrated [28], *nos-1* mRNA specifically labels PGCs, and its 3’-UTR stabilizes the transcript in PGCs but not in somatic cells. Therefore, with this approach, we could detect and quantify PGCs based on the GFP linked with *nos1* 3’-UTR in the construct used for mRNA preparation. To confirm that the PGC reduction is elicited only by the ablation of *ambra1b* paralog, we co-injected GFP-*nos1*-3’UTR mRNA and *ambra1a* or *ambra1b* mRNAs in one-cell stage embryos obtained by breeding of *ambra1b* heterozygous females and homozygous males. As expected, co-injection with *ambra1a* mRNA did not rescue PGC numbers in *ambra1b*^−/−^ embryos, which still displayed a 45% reduction of PGCs when compared to *ambra1b*^+/−^ control siblings (Fig. 3D, p = 0,0004). On the other hand, when injected with *ambra1b* mRNA, *ambra1b*^−/−^ embryos showed an almost complete recovery in the number of PGCs, with a residual loss of only 9% (Fig. 3E).

In addition, this experimental approach allowed to trace PGCs during development up to 72 hpf and revealed that PGC loss in *ambra1b*^−/−^ embryos is maintained at least until these developmental stages (Fig. 3F, 10 hpf: 43% reduction of PGCs, p = 0,0002; 24 hpf: 42% reduction of PGCs, p = 0,0008; 72 hpf: 46% reduction of PGCs, p = 0,0001). To understand whether Ambra1b is required for the correct development of germ cells only during the early developmental stages or also in later ones, we injected one-cell stage WT embryos with the *ambra1b*-splicing MO and let them grow to sexual maturity until 90 dpf. The resulting fish were 42% females and 58% males, indicating that knockdown of *ambra1b* transcripts early in development is not sufficient to prevent ovarian formation. This result suggests that the function of Ambra1b is not exclusively limited to the regulation of PGC number during early developmental stages, but it is also required at later stages.

### Human *AMBRA1* mRNA can rescue PGC number and involves interaction with the CUL4–DDB1 complex

Injection of *hAMBRA1* mRNA has already been proven to be effective in recovering the loss of Ambra1a and Ambra1b functions in previous studies in which MOs injection was used to down-regulate zebrafish *ambra1* isoforms [16, 17].

To further validate the rescue experiments performed in the present study, we verified that the *hAMBRA1* mRNA injected is indeed translated into protein. Two different transcripts, h*AMBRA1* [17] and h*AMBRA1*-RFP-sspB [8], both containing the entire coding region of *hAMBRA1*, were injected into one-cell stage WT embryos. Western blotting with antibody against hAMBRA1 using proteins extracted from 24-hpf embryos confirmed the translation of injected RNAs (Additional file 1, Fig. A2).

**Fig. 4:**
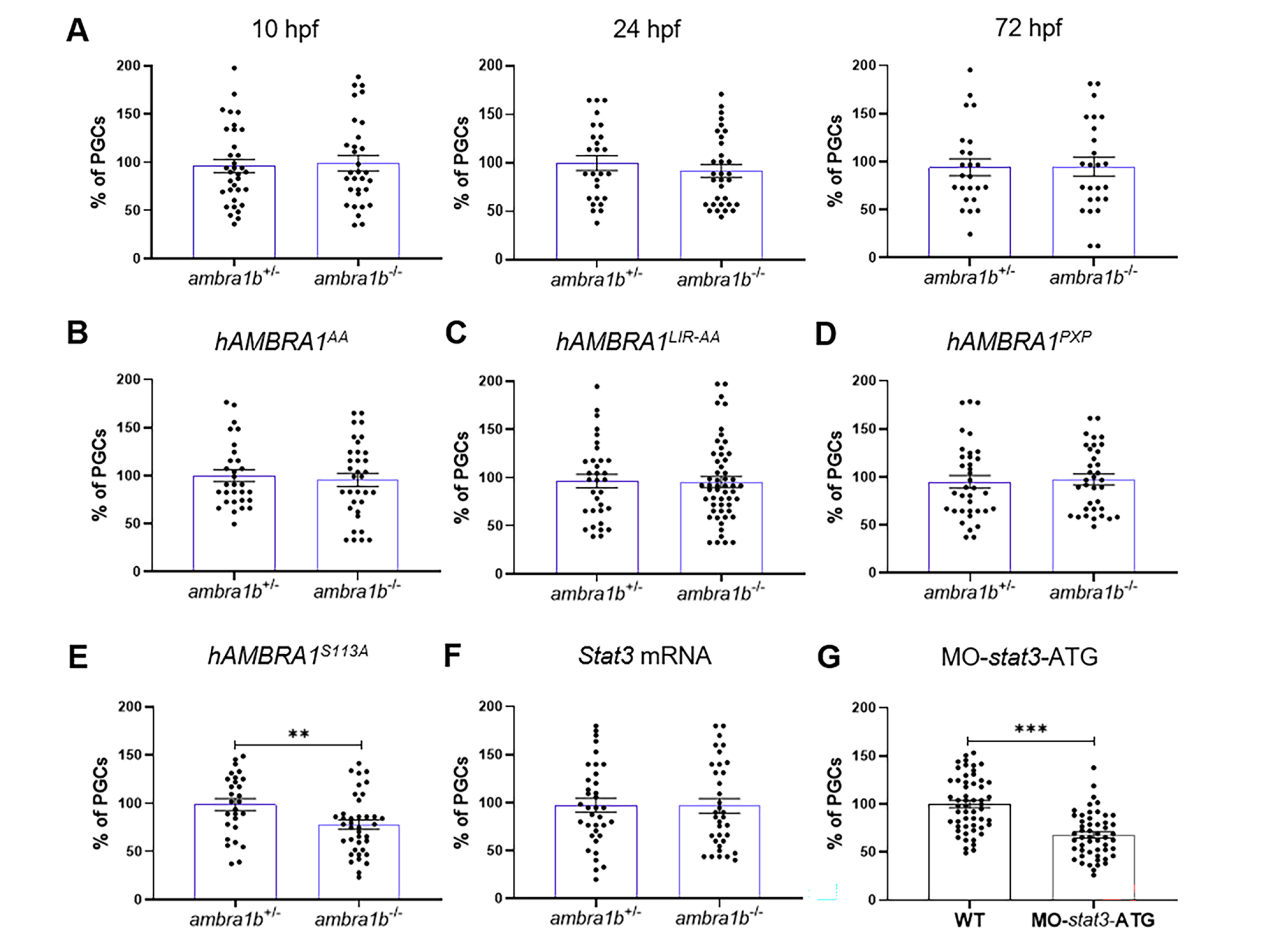
**Injection of *hAMBRA1*** **mRNA counteracts the loss of PGCs.** (**A**) Quantification of PGCs at 10, 24 and 72 hpf after co-injection of one-cell embryos with GFP-*nos1*-3’UTR and *hAMBRA1* mRNA (10 hpf: *ambra1b*^+/−^ n = 34, *ambra1b*^−/−^ n = 31; 24 hpf: *ambra1b*^+/−^ n = 26, *ambra1b*^−/−^ n = 32; 72 hpf: *ambra1b*^+/−^ n = 24, *ambra1b*^−/−^ n = 25). (**B-E**) Quantification of PGCs after injection with GFP-*nos1*-3’UTR mRNA and *hAMBRA1* mRNA mutated in TRAF6 (*hAMBRA1*^*AA*^, B), LC3 (*hAMBRA1*^*LIR −* AA^, C), PP2A (*hAMBRA1*^*PXP*^, D) and CUL4–DDB1 (*hAMBRA1*^*S113A*^, E) binding sites (B: *ambra1b*^+/−^ n = 30, *ambra1b*^−/−^ n = 34; C: *ambra1b*^+/−^ n = 33, *ambra1b*^−/−^ n = 54; D: *ambra1b*^+/−^ n = 36, *ambra1b*^−/−^ n = 35; E: *ambra1b*^+/−^ n = 28, *ambra1b*^−/−^ n = 39). (**F**) Injection of one-cell stage *ambra1b*^+/−^ and *ambra1b*^−/−^ embryos with murine *Stat3* mRNA and quantification of PGCs at 10-hpf (*ambra1b*^+/−^ n = 29, *ambra1b*^−/−^ n = 27). (**G**) Injection of one-cell stage WT embryos with MO-*stat3*-ATG and quantification of PGCs at 10-hpf (*ambra1b*^+/−^ n = 55, *ambra1b*^−/−^ n = 52). In all panels, the PGC number is expressed as percentage with respect to *ambra1b*^*+/−*^ animals, in which the PGC number was settled as 100%. The histograms show data pooled together from four (**A**, **F**) or three (**B**, **C**, **D**, **E**, **G**) independent experiments. Error bars indicate SEM. Statistical analysis was performed using Student’s t-test. ** *P* < 0.01, *** *P* < 0.001

Hence, we crossed female *ambra1b*^+/−^ with male *ambra1b*^−/−^ and injected the one-cell stage embryos thus obtained with both GFP-*nos1*-3’UTR and *hAMBRA1* mRNAs. Then we analyzed the number of PGCs at 10, 24 and 72 hpf (Fig. 4A). *hAMBRA1* mRNA effectively rescued the PGC number at 10 and 24 hpf, with no cell loss at 10 hpf and only 5% loss at 24 and 72 hpf (Fig. 4A). Moreover, we injected one-cell stage embryos with *hAMBRA1* mRNA and analysed the gonads at 45 dpf. The injected *ambra1b*^−/−^ fish did not show ovaries, even at 45 dpf, thus confirming that, although there were no significant differences in the PGC number at 3 dpf, a single injection of *hAMBRA1* cannot elicit a permanent rescue of the phenotype (Table 2). This result confirmed that Ambra1b is required for a prolonged period to assure the development of a functional ovary and that lack of this protein delays gonadal development.

**Table 2:** Histological analysis of 45-dpf zebrafish gonads after injection of *hAMBRA1* mRNA

	Undifferentiated gonad	Juvenile gonad	Juvenile ovary to testis transition	Ovary
*ambra1b* ^+/−^	-	-	2/8	6/8
*ambra1b* ^*−*/−^	1/8	3/8	4/8	-

#### Table 2

Summary of the data obtained with the histological analysis of 45-dpf *ambra1b*^+/−^ and *ambra1b*^−/−^ zebrafish siblings after injection of *hAMBRA1* mRNA. The numbers reported refer to the individuals presenting a specific type of gonad out of the total number of fish analysed.

AMBRA1 is involved in several biological processes through specific interactions with other proteins. The better-described interactors of AMBRA1 are LC3, TRAF6, CUL4–DDB1 and PP2A, which work as regulatory partners of AMBRA1 [1, 2, 5, 29]. To better understand how AMBRA1 acts in the regulation of PGC number in zebrafish, we injected in one-cell stage embryos four mutated forms of *hAMBRA1* mRNA which produce an AMBRA1 protein that cannot interact with either LC3 (*hAMBRA1*^*LIR − AA*^; [29]), TRAF6 (*hAMBRA1*^*AA*^; [1]), PP2A (*hAMBRA1*^*PXP*^; [5]) or CUL4–DDB1 (*hAMBRA1*^*S113A*^; [2]). Failure to bind LC3, TRAF6 or PP2A did not affect the *hAMBRA1*-dependent recovery of PGC number in *ambra1b*^*−*/−^ embryos (Fig. 4B-D), whereas *hAMBRA1*^*S113A*^ elicited only partial rescue of PGC number (Fig. 4E, 49% reduction of PGCs, p = 0,010), suggesting that AMBRA1-CUL4–DDB1 interaction is required for PGC maintenance.

A recent study has shown a relationship among AMBRA1, CUL4–DDB1 complex, and STAT3 in medulloblastoma (MB) stem cells, demonstrating that AMBRA1, through its direct interaction with the CUL4–DDB1 complex, is involved in the degradation of SOCS3, an inhibitor of STAT3 activity [30]. In this way, up-regulation of AMBRA1 activates STAT3 and leads to enhanced stem potential, whereas knockdown of *AMBRA1* reduces MB stem cell growth and migratory potential.

Based on these findings, we assessed the involvement of STAT3 in the regulation of zebrafish PGCs by providing murine *Stat3* mRNA to *ambra1b* KO embryos, as well as by injecting a zebrafish *stat3* ATG-MO to block the translation of maternal and zygotic *stat3* transcripts in WT embryos. Remarkably, injection of murine *Stat3* mRNA was able to completely recover the PGC loss of 10 hpf *ambra1b* KO embryos (Fig. 4F), whereas injection of zebrafish *stat3* MO in WT embryos determined a significantly decreased PGC number when compared to non-injected WT embryos (Fig. 4G, 32% reduction of PGCs, p = 0,001). These results point at the Ambra1b-Cul4-Stat3 molecular pathway in the regulation of PGC number during the first zebrafish developmental stages.

### *Ambra1* is highly expressed in mouse ovary and its haploinsufficiency affects gene expression and follicle maturation

**Fig. 5:**
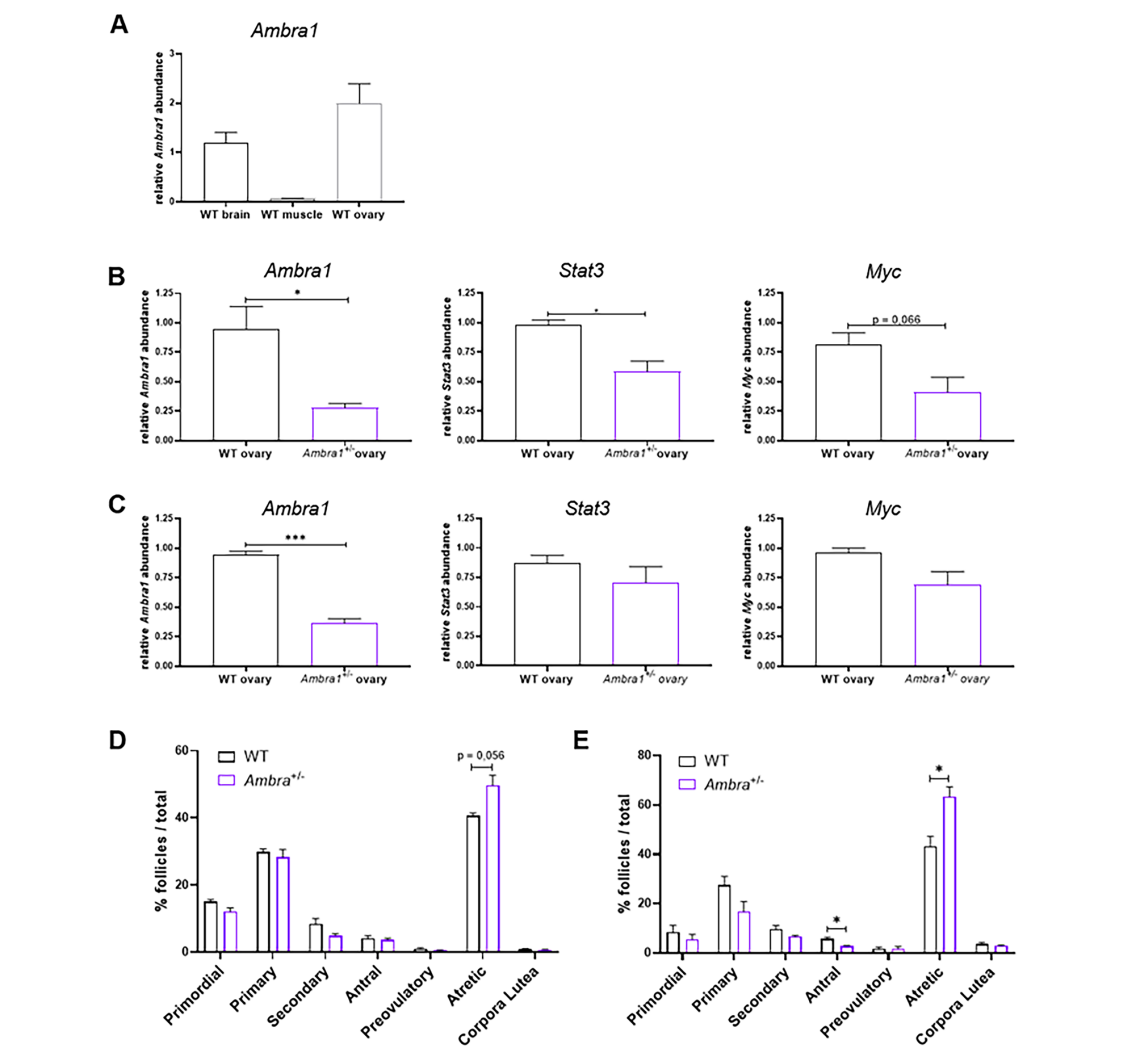
**Ambra1 deficiency affects ovary physiology in mice.** (**A**) RT-qPCR analysis of Ambra1 mRNA levels in brain, muscle and ovaries of 3-month-old WT mice. (**B**, **C**) RT-qPCR analysis of *Ambra1*, *Stat3* and *Myc* expression in 3-month-old (**B**) and 10-month-old (**C**) *Ambra1*^+/+^ (WT) and *Ambra1*^+/−^ sibling mice. Data were generated from three mice of each genotype for each time point. Values represent the mean ± SEM. Data were normalized with the housekeeping gene *Gapdh*. Statistical analysis was performed using Student’s t-test. *, *P* < 0.05. (**D**, **E**) Comparison of follicle proportion in ovaries of 3-month-old (**D**) and 10-month-old (**E**) *Ambra1*^+/+^ and *Ambra1*^+/−^ mice. The proportion of follicles is displayed as the percentage of each follicle type over the total number of follicles (primordial, primary, secondary, antral, preovulatory, atretic and corpora lutea) over the total number of follicles. Values represent the mean ± SEM. Data were generated from three mice of each genotype for each time point, except for 3-month-old *Ambra1*^+/−^ mice (four ovaries). Statistical analysis was performed using Student’s t-test. *, *P* < 0.05

Since the above results showed the ability of the *hAMBRA1* mRNA to recover the number of PGCs in mutant *ambra1b*^−/−^ zebrafish, we carried out further work aimed at assessing the role of Ambra1 in the differentiation of mice ovaries. Similar to zebrafish, *Ambra1* showed high levels of expression in 3-months-old mouse ovaries (Fig. 5A). We then assessed the expression of *Ambra1*, *Stat3*, and *Myc* (one STAT3 target gene) at three and ten months of life, using ovaries from *Ambra1*^+/−^ and *Ambra1*^+/+^ sibling mice (Fig. 5B,C). All heterozygous ovaries showed a significant downregulation of *Ambra1* transcripts (Fig. 5B, 3 months, p = 0,0165; Fig. 5C, 10 months, p = 0,0002) and a general reduction of the expression levels of *Stat3* and *Myc*. The first was found to be statistically significant at three months (Fig. 5B, p = 0,0154), pointing at an Ambra1-related function for STAT3 in this organ.

The proportion of the different types of follicles in the ovaries of *Ambra1*^+/+^ and *Ambra1*^+/−^ sibling mice did not show significant differences at three months of age (Fig. 5D). However, a significant reduction of antral follicles in parallel with a significant increase in atretic follicles was found at 10 months, thus pointing at a role for Ambra1 in mouse follicolar development regulation (Fig. 5E, antral follicles p = 0,0213; atretic follicles p = 0,0232).

## Discussion

AMBRA1 is a well-known protein in the biological landscape, whose biochemical characteristics, presence of specific domains and stretches of intrinsically disordered structure, allow interaction with several proteins, thus regulating a plethora of different functions, as demonstrated in mammalian cell lines and mouse models [1, 2, 4–7, 9, 10].

In zebrafish, Ambra1 is encoded by two paralogous genes, for which we recently generated two knockout zebrafish lines using the CRISPR/Cas9 technology [17]. Unlike the results previously obtained with knockdown MOs approaches, in which silencing of both paralogs led to severe phenotypes, the activation of genetic compensation mechanisms in stable mutant lines led to the lack of overt phenotypes, at least during the developmental stages [17]. Nevertheless, in agreement with the persistence of duplicated genes only after acquisition of new functions, sub-functioning of ancestral ones or tissue and/or temporal-specific expression, we found that the two *ambra1* genes show different tissue-specific expression in the adult districts we analysed. Moreover, the lack of *ambra1b*^*−/−*^ female individuals, together with the high level of expression of both transcripts in gonads and particularly in the ovary, pointed to a significant role of Ambra1 in gonadal development, physiology and pathology.

Since the number of germ cells represents a critical factor in zebrafish sex determination, we analysed the PGC number in our mutant lines and found that *ambra1b* KO causes a severe reduction of these stem cells, thus suggesting a critical role in PGC survival. To confirm these data, we adopted different experimental approaches, starting with ATG and splicing MOs, which have already been validated for both paralogous genes [15–17]. These experiments confirmed that the protective function of PGCs is limited to *ambra1b*, whereas *ambra1a* silencing does not affect PGC number. In addition, although we cannot rule out the role of maternal *ambra1b* mRNAs, the results we obtained with splicing MOs suggest that proteins of embryonic origin may have an even more critical role in this process. Rescue experiments added further validations to the exclusive role of Ambra1b, since only *ambra1b* transient overexpression hampers the loss of PGCs.

Moreover, rescue experiments with the *hAMBRA1* transcript led to a complete recovery of the PGC number, suggesting that this function, or the protein interaction on which it is based, is evolutionary conserved. Since AMBRA1 can interact with several proteins involved in different cellular functions, we replicated rescue experiments using *hAMBRA1* mRNA mutated in specific binding sites for known AMBRA1 interacting partners. The results showed that only the interaction with the CUL4-DDB1 complex is unable to fully recover PGC number in *ambra1b*^*−/−*^ embryos, thus suggesting that the protective function of Ambra1b relies on the interaction with this complex. The CUL4-DDB1 ubiquitin ligase complex can regulate a wide range of cellular processes through interaction with substrate receptors (called DCAFs, DDB1 and CUL4-associated factors) and ubiquitination of specific target proteins [31]. AMBRA1, also known as DCAF3, is one of the WD40 repeat-containing proteins that can act as substrate receptor [6].

Interestingly, Cul4b, a member of the CUL4-DDB1 complex, initially studied in *C. elegans* where it is involved in the replication of DNA [32], was found to be fundamental for the maintenance of germ cells in the testes of *D. melanogaster* [33]. Moreover, in mouse, it has both a cell-autonomous and a non-cell-autonomous function in testis physiology, as it is required for male germ cells spermatogenesis, and in somatic cells to maintain the spermatogonial stem cell population in the testis [34].

The functions of this complex in mouse ovary physiology and oocyte survival have been analysed by means of oocyte-specific KO of DDB1, the linker between CUL4 and the DCAF proteins. The silencing of this protein, as well as deletion of DCAF1, determines oocyte loss and ovarian insufficiency [35]. However, other DCAFs are involved in gonadal physiology: DCAF13 was found to regulate ovarian follicle maintenance and oocyte development and growth [36], while DCAF8 and DCAF17 have critical roles in spermatogenesis [37, 38].

AMBRA1 binds CUL4-DDB1 complex and targets D-type cyclins for ubiquitin-mediated degradation, thus acting as a tumor suppressor and assuring normal cell cycle progression [9, 39]. Whereas regulation of this process does not correspond to a protective role from germ cell loss, interaction of AMBRA1 with the CUL4-DDB1 complex allows for degradation of SOCS3, an inhibitor of STAT3 activity [40]. STAT3 is a fundamental transcription factor implicated in the proliferation and migration of stem cells, including those of medulloblastoma [30]. Hence, considering the role of STAT3 in stem cell homeostasis [41, 42], we sought to assess whether Ambra1 role in PGCs is related to STAT3.

Knockdown experiments with a *stat3* ATG-morpholino resulted in a significant reduction in PGC number at 10 hpf, suggesting the involvement of Stat3 in the regulation of PGC number in zebrafish. This hypothesis is also supported by the rescue of PGC number after injection with the murine *Stat3* mRNA. To validate this interaction, we analysed *Ambra1*, *Stat3* and *Myc* expression in the ovary of *Ambra1*^*+/+*^ and *Ambra1*^*+/−*^ mice. *Ambra1* was found highly expressed in WT ovaries and reduced in heterozygous samples. Interestingly, in heterozygous mouse ovaries the expression of *Stat3* and *Myc*, was also reduced, although significantly only at three months of age for *Stat3*. Moreover, analysis of the different types of follicles showed a significant increase in atretic follicles and a reduction of the antral ones in *Ambra1*^*+/−*^ mice at 10 months of age. Sex determination is driven by different mechanisms in mammals and in zebrafish. Indeed, murine sexual differentiation is genetically determined, whereas in zebrafish it is settled by a combination of genetic and environmental conditions. However, an excessive loss of germ cells is associated with reproductive problems in mammals [43] and therefore the results we obtained in mice support a possible role for Ambra1 in the quality control and fertility capabilities of female mice. Future insights into Ambra1 role in gonadal development and physiology may help uncover new aspects of male and female infertility, a pathological problem in which many cases are still considered idiopathic.

The high level of expression of both paralogs in the gonads suggests the involvement of these proteins in other aspects of reproductive physiology. In anticipation of more in-depth studies in this field, we analysed the reproductive performance of both sexes, taking advantage of the *ambra1b*^−/−^ females derived from the single mutant escaper we found up to now, and found a significant impairment of the reproductive process in all cases. Notably, the administration of the probiotic *L. rhamnosus* to zebrafish females increases the expression of genes involved in the autophagic process, including *ambra1b*, and downregulates transcripts related to apoptosis, thus modulating the balance between the two processes and regulating ovarian functions [44].

Finally, in agreement with the AMBRA1 role as a tumor suppressor, among the mutant males we analysed we found two cystic degenerations of the seminiferous tubules, a pre-cancer condition, two seminomas and an undifferentiated germ cell tumor. Although testicular lesions such as seminomas and cystic degeneration of seminiferous tubules with or without hyperplasia of spermatogones are common changes in adult zebrafish [45], seminomas were found to be less than 2% in a survey of nearly 10,000 2-year-old zebrafish [46] and 17% in WT zebrafish of 30–34 months of age [47]. Since we did not detect seminomas or undifferentiated germ cell tumors in any WT, the higher incidence of tumors in the testis of mutants suggests a key role of Ambra1a and Ambra1b as tumor suppressors in this organ.

## Conclusion

In conclusion, by exploiting *ambra1a* and *ambra1b* KO zebrafish lines, we were able to prove the sub-functionalization between these two genes and uncover a novel function of Ambra1 in the protection from excessive PGC loss, which seems to require binding with the CUL4-DDB1 complex. Since both Ambra1a and Ambra1b proteins contain the binding domain for CUL4-DDB1, but only Ambra1b is involved in PGC maintenance, it can be speculated that this interaction requires an additional functional domain that is present in the human AMBRA1 but not in the zebrafish Ambra1a isoform. Further work in the near future may allow to dissect the molecular basis of this interaction together with a more precise identification of all actors involved, as well as of the roles played by the two Ambra1 isoforms in ovarian and testicular physiology.

## Materials and methods

### Animal maintenance and handling

Zebrafish embryos, larvae, and adults were maintained according to standard procedures [48]. Embryos were obtained from natural spawning and raised at 28.5 °C in a 12:12 light:dark (LD) cycle in fish water (50X: 25 g Instant Ocean, 39.25 g CaSO_4_ and 5 g NaHCO_3_ for 1 L). All husbandry and experimental procedures complied with the Italian and European Legislation for the Protection of Animals used for Scientific Purposes (Directive 2010/63/EU) and were approved by the Animal Ethics Committee of the University of Padua and by the Italian Ministry of Health (Authorization Number 568/2016-PR). The experiments described in this paper were performed with wild-type (WT) zebrafish or with the *ambra1a* and *ambra1b* mutant lines whose generation has been previously described [17]. Non-mutant fish indicated as WT and *ambra1a*^*+/+*^*/ambra1b*^*+/+*^ correspond to animals deriving from different or the same batches of the knockouts, respectively. However, the genetic background of the WT fish was the same used for the generation of the *ambra1a* and *ambra1b* mutant lines.

Heterozygous whole-body *Ambra1* knockout (*Ambra1*^+/–^) mice [10] were housed in controlled temperature (23 °C) and light (12:12 light:dark cycle) conditions, with free access to water and food. Animal procedures were approved by the Animal Ethics Committee of the University of Padua and by the Italian Ministry of Health (Authorization Number 581/2017-PR). All animals were in C57BL/6 N genetic background. *Ambra1*^*+/–*^ mice and the age-matched littermate controls *Ambra1*^+/+^ were sacrificed by cervical dislocation. Ovaries were dissected, isolated and immediately frozen in liquid nitrogen vapours or fixed in Bouin’s solution for further experiments.

### DNA extraction and genotyping

Biopsies from the caudal fin of larvae and adult fish were used for fin clips genomic DNA extraction performed with the HotSHOT protocol [49]. For the screening of each genotype, fragments at the target sites were amplified by PCR and the locus-specific primers [17] are reported in in the Additional file 2, Table A1.

Mice genotyping was determined by PCR from digested ear biopsies with specific primers for *Ambra1* knockout allele and *Ambra1* wild-type allele [10] (Additional file 2, Table A1).

### Morphological and histological analyses

Mutant and WT fish, heterozygote, and WT mice ovaries were fixed for 24/48 h in Bouin’s solution at room temperature. Samples were dehydrated through a graded ethanol series, infiltrated with xylene, and embedded in Paraplast plus (Leica Biosystem, 39,602,004). Samples were serially cut into 7–8 μm sections on an LKB microtome. After rehydration, sections were stained with hematoxylin and eosin and mounted with Eukitt (BioOptica, 09–00100) for microscopy examination.

### Morpholinos injection

Morpholino (MO) (Gene Tools) treatment was performed with MOs against the ATG translation initiation sites of either *ambra1a* or *ambra1b* transcripts (MO-*ambra1a*-ATG and MO-*ambra1b*-ATG) and with splice-blocking MOs designed at the exon 3/intron 3 junction sequence of both genes (MO-*ambra1a*-splice and MO-*ambra1b*-splice). All MOs were previously described and validated [15–17]. A MO against the ATG translation initiation sites of *Stat3* (MO-*stat3*-ATG) was also used, as previously described and validated [50]. For each *ambra1* MO, 8.2 ng were injected in the yolk of one-cell stage embryos whereas 10 ng were injected for the MO-*stat3*-ATG. Injections were performed under a dissecting microscope using a microinjector (Leica Microsystems).

### Vasa immunohistochemistry

The number of PGCs was analysed in WT, knockout (KO), and knockdown (KD) 10-hpf zebrafish embryos by whole-mount immunohistochemistry with an anti-Vasa antibody (1:5000 polyclonal rabbit) [51] kindly provided by Prof. Knaut (New York University School of Medicine, USA). PGCs were manually counted by visualization in brightfield with Leica M165 FC microscope equipped with a Nikon DS-Fi2 digital camera.

### GFP-*nos1*-3’UTR mRNA synthesis and injection

PGCs were visualized with fluorescence microscopy by injection of mRNA produced using the construct GFP-*nos1*-3’UTR [27], kindly provided by Prof. Raz (University of Münster, Germany). The mRNA was transcribed with the SP6 promoter and the mMessage Machine kit (Ambion) according to manufacturer’s instruction, after plasmid linearization with *Not*I restriction enzyme (Promega).

For rescue experiments, cDNAs of human *AMBRA1* (*hAMBRA1*), as well as *hAMBRA1* mutated in the PP2A, LC3, TRAF6 and CULLIN4 binding sites (*hAMBRA1*^*PXP*^, *hAMBRA1*^*LIR−AA*^, *hAMBRA1*^*AA*^ and *hAMBRA1*^*S113A*^) were inserted in pCS2 + plasmids as stated in a previous paper [17] or produced for this study. mRNAs were then transcribed using the T3 promoter and the mMessage Machine kit, after plasmid linearization with *Hind*III restriction enzyme (Promega). Zebrafish *ambra1a1* and *ambra1b* were inserted in pCS2 + plasmids and transcribed using the SP6 promoter and the mMessage Machine kit, after plasmid linearization with *Not*I restriction enzyme (Promega). All mRNAs were polyadenylated at the 3’-termini using a Poly(A) Tailing Kit (AM1350; Invitrogen). The mRNAs were injected in one-cell stage embryos at the concentration of 80 pg per embryo for the GFP-*nos1*-3’UTR mRNA and 40 pg per embryo for the various *hAMBRA1* mRNAs. AMBRA1 mutants in LC3, TRAF6, CUL4-DDB1 and PP2A binding sites were generated by using the site-directed mutagenesis kit (Agilent Technologies) respectively. The sequences used are as reported in [1, 2, 5, 29]. The murine Stat3 mRNA (*mStat3*) was also prepared as described [52] and injected in one-cell stage embryos at the concentration of 50 pg.

### Protein extraction and western blotting

Pools of 50 WT embryos (24 hpf) were injected with *hAMBRA1* mRNA and *hAMBRA1*-RFP-sspB mRNA [8, 17], deyolked with Ringer’s solution and frozen in liquid nitrogen. For the protein extraction, samples were added with protease inhibitors (Roche, COEDTAF-RO) and mechanically homogenized in Tissue Extraction Reagent I (Invitrogen, FNN0071). Following brief centrifugation, the supernatant was collected, and the protein content was determined by BCA assay (Thermo-Fisher Scientific, 23,225). 30 µg of total proteins per sample were boiled for 10 min at 90 °C and loaded into 12% polyacrylamide Novex NuPAGE Bis-Tris gels (Thermo- Fisher Scientific, NP0341). Following SDS-PAGE, proteins were transferred onto a PVDF membrane (Millipore, IPVH00010). This was subsequently saturated for 1 h at RT with 5% non-fat dry milk (Bio-Rad, 1,706,404) in 1X Tris-Buffered Saline added with 0.1% Tween20 detergent (Sigma-Aldrich, P9416) and then hybridized O/N at 4 °C with primary antibodies (anti-hAMBRA1 1:1000, Thermo-Fisher Scientific, PA1-16930; anti ACTB/β-actin 1:2000, Sigma-Aldrich, A5316) and for 1 h at RT with secondary antibodies (HRP-conjugated goat anti-rabbit, Bethyl Laboratories Inc.; anti-mouse IgG-Fc fragments, A120-111P and A90-131P). The chemiluminescent signal was revealed with SuperSignal West Pico Chemiluminescent Substrate (Thermo-Fisher Scientific, 340,779). Densitometric quantification was carried out by FIJI software.

### RNA extraction, reverse transcription and qPCR

Total RNA was extracted using Quick-RNA Miniprep kit (Zymo Research R1054) from single ovaries of 3- and 10-months-old WT and heterozygote mice, and from organs of 6 mpf WT zebrafish (brain, intestine, liver, muscle, ovary, testis). RNA samples were kept at -80 °C until use. 5 µg of the total RNA obtained from mice ovaries were used for mRNA isolation with Dynabeads mRNA DIRECT Kit (00460456; Invitrogen). All the mRNA obtained with this procedure as well as 1 µg of the total RNA obtained from zebrafish samples was used for cDNA synthesis, employing FIREScript Reverse Transcriptase Kit (06-13-00050; Solis BioDyne) and following the manufacturer’s protocol. Quantitative polymerase chain reaction (qPCR) was performed with SYBR green method on Biorad cfx384 (Biorad). The reaction conditions were as follows: enzyme activation at 95 °C for 15 min followed by 45 cycles of denaturation (30 s at 95 °C), annealing (30 s at 60 °C), and extension (20 s at 72 °C). Fluorescence monitoring occurred at the end of each cycle. *Gapdh* (mice samples) and *actb2* (zebrafish samples) mRNAs were used as normalizers. The primer sequences are reported in the Additional file 2, Table A1.

### Zebrafish gonads *in situ* hybridization

Ovaries and testicles extracted from 6-mpf WT zebrafish were fixed overnight at 4 °C with PFA 4% in PBS added with 0.1% Tween-20 and 0.1% DMSO and then stored in 100% methanol at -20 °C until used. For *in situ* hybridization, samples were rehydrated through a graded series of methanol in PBT (PBS and 0.1% Tween-20), permeabilized with proteinase K for 30 min at room temperature and fixed again with PFA 4% in PBT for 30 min at room temperature. Fixation was stopped with several brief washes in PBT; then organs were prepared for *in situ* hybridization as previously described [15]. Briefly, ovaries and testis were prehybridized for 2–5 h at 65 °C and then incubated with specified probes at 65 °C overnight. Hybridized probes for the *ambra1a* and *ambra1b* transcripts [15] were detected with sheep anti-digoxigenin-AP Fab fragments (Roche Diagnostics, Mannheim, Germany) and visualized with the chromogen substrate NBT/BCIP Stock solution (Roche Diagnostics, Mannheim, Germany). The chromogenic reaction was stopped by PBT rinsing followed by a final fixation in 4% PFA in PBS overnight at 4 °C. Samples were dehydrated in a graded series of methanol, then cleared in a clearing solution (2:1 benzyl benzoate:benzyl alcohol), and finally rehydrated to PBS. Organs were equilibrated in a graded series of glycerol for image acquisition.

### Zebrafish reproductive performance analysis

The reproductive performance of *ambra1a*^−/−^ and *ambra1b*^−/−^ males and females was assessed by natural reproductions in spawning tanks under standard aquarium conditions (Westerfield 1995). Four 8-mpf animals were analysed for each sex and genotype. WT partners were randomly chosen each time between the batch of WT males and females (housed separately from the opposite sex). Animals underwent 4 consecutive reproductive rounds, once every 10 days, to ensure we had a realistic mean of the reproductive performances of each individual. We excluded a specific male-female interaction effect by performing repeated reproductive rounds for each animal coupled with different WT partners. At the end of each trial, spawned eggs were collected in a Petri dish with fish water, following the standard husbandry rules [48]. Reproductive performances were quantified as the number of times in which the male was able to induce the female to spawn on the total 4 chances. Considering the successful trials, it was evaluated the mean number of eggs spawned, the mean fertilization rate (fertilised eggs/total eggs) and the mean offspring survival rate at 6 dpf [53].

### Mice ovaries follicle count

An equal number of ovaries from WT and *AMBRA1*^*+/−*^ heterozygous mice were scored for each age point (3 and 10 months). The scoring system was set up following the methods proposed by previous literature [54, 55].

Briefly, the total follicle number was estimated by counting and classifying all the follicles in every 9th section of the ovary. Only follicles in which the nucleus was visible were included in the count. The total follicle number was then calculated by multiplying the raw counts per 9 [54, 55]. Counts were collected in customized Excel files, and the total and estimated numbers of follicles for each category were calculated. To allow for comparison between ovaries of different sizes we used the percentage of each category instead of the total number. For the accurate estimation and correct classification of ovarian follicles, the recommendations of Myers and collaborators were followed [56]. Representative examples are provided in Additional file 1, Fig. A3.

### Imaging

For imaging of PGCs at 10 and 24 hpf, embryos were anaesthetized with 0.04% tricaine and mounted in 2% methylcellulose on a depression slide. Images of PGCs at these embryonic stages were recorded with a Leica DMR using a Nikon DS- Fi2 digital camera. Three dpf embryos were instead embedded in 0.8% low-melting agarose and analysed with the Nikon C2 confocal system provided with the software NIS ELEMENTS.

WMISH-stained organs, as well as immunohistochemistry embryos, were mounted in 80% glycerol in phosphate-buffered saline plus 0.1% Tween 20 (Sigma, P1379), observed under a Leica M165 FC microscope, and photographed with a Nikon DS-Fi2 digital camera. Histological samples were photographed on a Leica DMR using a Nikon DS- Fi2 digital camera.

### Statistical analysis

Statistical analysis was performed with Graph Pad Prism V9.0.1. Data are presented as the means ± SEM. Comparisons between WT and KO/KD zebrafish/mice were performed with the tests reported on the figure captions. The p-values are indicated with the following symbols: *, p < 0.05; **, p < 0.01; ***, p < 0.001; ****, p < 0.0001.

## Electronic supplementary material

Below is the link to the electronic supplementary material.

Supplementary Material 1

Supplementary Material 2

## Data Availability

Mutant lines are available upon request. Supplemental information including three figures can be found in Additional file 1. All primers used in this study are listed in Additional file 2, Table A1.

## Abbreviations

AMBRA1: Activating molecule in beclin-1-regulated autophagy protein 1
CUL4-DDB1: Cullin4-DNA damage binding protein 1 complex
DCAF: DDB1- and CUL4-associated factor
dpf: Days post fertilization
GFP: Green fluorescent protein
hAMBRA1: Human AMBRA1
hpf: Hours post fertilization
KD: Knockdown
KO: Knockout
LC3: Microtubule-associated protein 1 A/1B-light chain 3
LD: Light:dark
MB: Medulloblastoma
MO: Morpholino
mpf: Months post fertilization
mStat3: Murine Stat3
Myc: Myc proto-oncogene protein
nos1: Nanos1
PGCs: Primordial germ cells
PP2A: Protein phosphatase 2 A
RT-qPCR: Real-time quantitative PCR
SOCS3: Suppressor of cytokine signaling 3
STAT3: Signal transducer and activator of transcription 3
TRAF6: TNF receptor-associated factor 6
WT: Wild type
