# Supplementary material for: Zebrafish ambra1b knockout reveals a novel role for Ambra1 in primordial germ cells survival, sex differentiation and reproduction
Source: Biol Res. 2023 Apr 28;56:19. doi: 10.1186/s40659-023-00430-9 (PMC10142490; doi:10.1186/s40659-023-00430-9)
Supplement: Supplementary file 1 — Supplementary Material 1 [file 40659_2023_430_MOESM1_ESM.pdf]

Figure A1

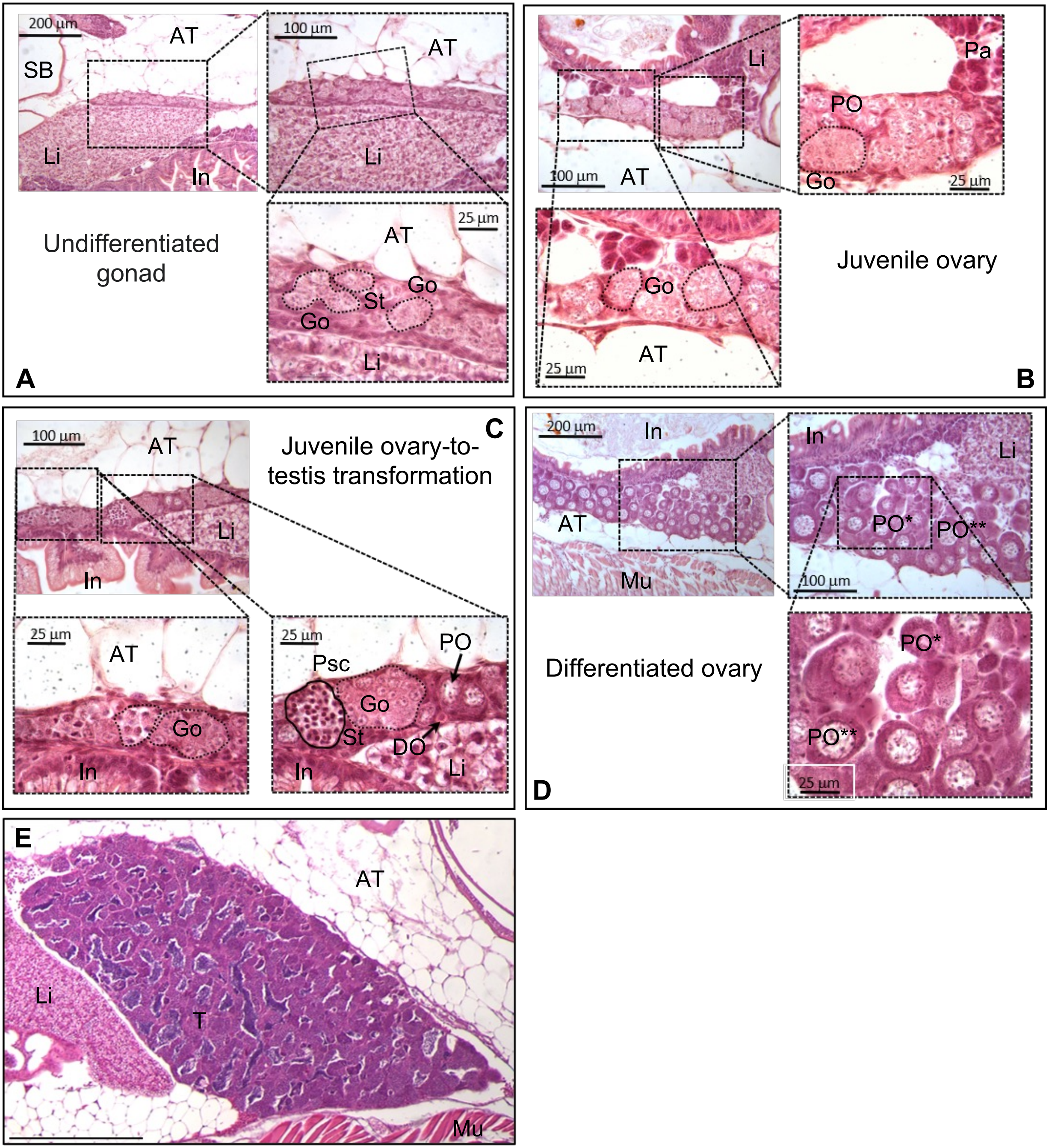

200 µm
AT
SB
Li
In
100 µm
AT
Li
25 µm
AT
Go
St
Go
Li
Undifferentiated gonad
A
Li
100 µm
AT
Pa
PO
Go
25 µm
Go
AT
25 µm
Juvenile ovary
B
100 µm
AT
Li
In
Juvenile ovary-to-testis transformation
C
25 µm
AT
Go
In
25 µm
PO
Psc
Go
St
DO
In
Li
200 µm
In
AT
Mu
In
Li
PO*
PO**
100 µm
Differentiated ovary
PO*
PO**
25 µm
D
E
AT
Li
T
Mu

Figure A1. Histological analyses of 35-dpf (A-D) and 60-dpf (E) zebrafish gonads. (**A**) Representative images of an undifferentiated gonad. The images highlight the elongated but thin shape of this structure, generally formed by a few layers of gonocytes, grouped into cysts (highlighted by the dashed outline). (**B**) Representative images of a juvenile ovary. The structure is elongated and consists of groups of gonocytes (highlighted by the dashed outline), among which some primary oocytes can be identified. (**C**) Representative images of juvenile ovary-to-testis transition. It is possible to observe the simultaneous presence of undifferentiated gonocytes (highlighted by the dashed outline), primary oocytes (first stage of oocyte development), degenerating oocytes and primary spermatocytes (highlighted by the continuous outline). (**D**) Differentiated ovary. It is possible to distinguish primary oocytes, at early (PO*) and more advanced developmental stages (PO**, late). (**E**) Representative image of 60-dpf *ambra1b*$^{-/-}$ mutant testis. AT = adipose tissue; DO = degenerating oocyte; Go gonocytes; Li = Liver; In = Intestine; Mu =muscle; Psc = primary spermatocytes; St= stroma; Pa= Pancreas; PO = primary oocytes; SB= swim bladder.

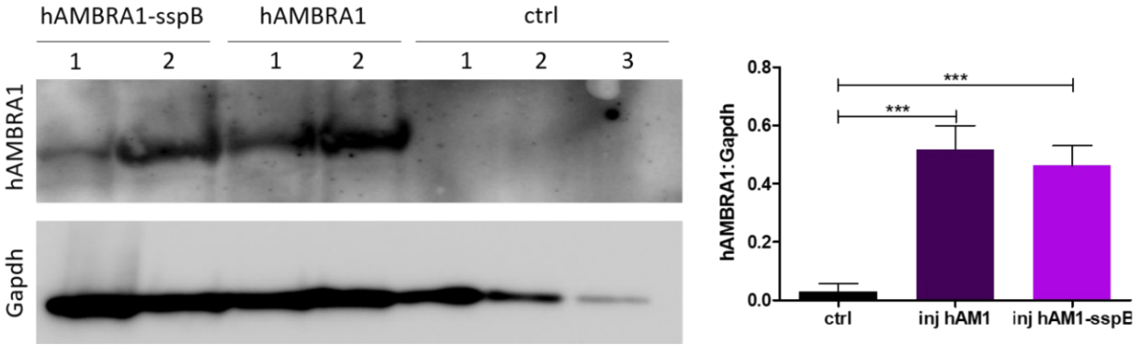

Figure A2. Western blot analysis with proteins extracted from un-injected WT embryos (ctrl), WT embryos injected with *hAMBRA1* (*hAM1*) and *hAMBRA1-RFP-sspB* (*hAM1-sspB*) mRNA at 24 hpf. Gapdh was used as control for protein loading. This experiment was performed two times, each time with two replicates. Graph reports densitometric analysis of band intensities (Injection with *hAM1*, *hAM1-sspb* n=4; ctrl n=6). Error bars indicate SEM. Statistical analysis was performed using Student's t-test. *** P < 0.001.

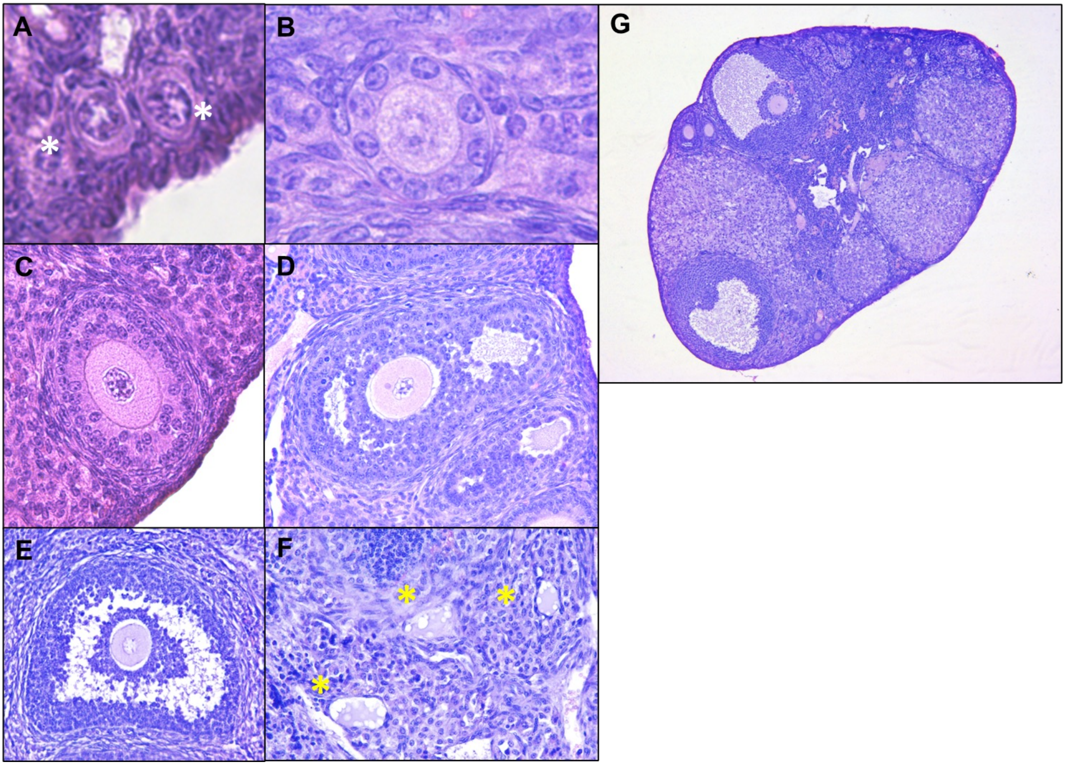

Figure A3. Representative stages used for the ovarian follicles counts. (**A**) Primordial follicles (white asterisks); (**B**) Primary follicle; (**C**) Secondary follicle; (**D**) Antral follicle; (**E**) Preovulatory follicle; (**F**) Atretic bodies (yellow asterisks); (**G**) Multiple corpora lutea.
