# Supplementary material for: Zebrafish ambra1b knockout reveals a novel role for Ambra1 in primordial germ cells survival, sex differentiation and reproduction
Source: Biol Res. 2023 Apr 28;56:19. doi: 10.1186/s40659-023-00430-9 (PMC10142490; doi:10.1186/s40659-023-00430-9)
Supplement: Supplementary file 2 — Supplementary Material 2 [file 40659_2023_430_MOESM2_ESM.pdf]

Species	Use	Gene	Sequence	Accession number
Mus musculus	RT-qPCR	*Gapdh-F*<br>*Gapdh-R*	5'-ATCCTGCACCACCAACTGCT-3'<br>5'-GGGCCATCCACAGTCTTGTG-3'	NG_047186.1
		Ambra1-F<br>*Ambra1-R*	5'-CTGCCTGATAGTCCACGCTC-3'<br>5'-TGTGTGGATGCCAAGAGAGTC-3'	NM_172669
		Stat3- F<br>*Stat3-R*	5'-CGAAAGTCAGGTTGCTGGTC-3'<br>5'-CTCTGAGGGCAGCAACATC-3'	NM_011486
		c-Myc-F<br>*c-Myc R*	5'-AGGCGGACACACAACGTCTT-3'<br>5'-TGCTCGTCTCTTGAATGGA-3'	NM_001177353.1
	Genotyping	*Ambra1 KO-F*<br>*Ambra1 KO-R*	5'-TGATAGTCCACGCTCGACCT-3'<br>5'-TGAACATTCCAGCTTGGTGC-3'	NM_172669.3
		Ambra1 WT-F<br>*Ambra1 WT-R*	5'-TCTGGTTGCCTAGATGGGGA-3'<br>5'-ACTCATGTTAGAGCCTCCTGC-3'	NM_172669.3
Danio rerio	RT-qPCR	*atb2-F*<br>*atb2-R*	5'-TGGGTATGGAATCTTGCGGT-3'<br>5'-GTGGGGCAATGATCTTGATC-3'	NM_181601.5
		ambra1a-F<br>*ambra1a-R*	5'-TCTGTGGGACTGGAGTCG-3'<br>5'-AGCGCTGTCGGAGATGAG-3'	NM_001281992
		ambra1b-F<br>*ambra1b-R*	5'-AGGTGACGGACAGTCAGC-3'<br>5'-CCTACCATCACATAGCAGC-3'	NM_001302220
	Genotyping	*ambra1a-F*<br>*ambra1a-R*	5'-TGGCTGCCTGGATGGAGAG-3'<br>5'-GGTGCAAAAGTTGCCGTAAT-3'	NM_001281992.1
		ambra1b-F<br>*ambra1b-R*	5'-GAAACTCTGTGCTGATCCTGT-3'<br>5'-GGCTTTAACAATTATATGCTGC-3'	NM_001302220.1

Table A1: Mice and zebrafish primers used in this work.
